# Failure of non-invasive ventilation in patients with acute lung injury: observational cohort study

**DOI:** 10.1186/cc4923

**Published:** 2006-05-12

**Authors:** Sameer Rana, Hussam Jenad, Peter C Gay, Curtis F Buck, Rolf D Hubmayr, Ognjen Gajic

**Affiliations:** 1 Department of Internal Medicine, Division of Pulmonary and Critical Care Medicine, Mayo Clinic, Rochester, Minnesota USA; 2 Department of Anesthesiology, Division of Intensive Care and Respiratory Care, Mayo Clinic, Rochester, Minnesota USA

## Abstract

**Introduction:**

The role of non-invasive positive pressure ventilation (NIPPV) in the treatment of acute lung injury (ALI) is controversial. We sought to assess the outcome of ALI that was initially treated with NIPPV and to identify specific risk factors for NIPPV failure.

**Methods:**

In this observational cohort study at the two intensive care units of a tertiary center, we identified consecutive patients with ALI who were initially treated with NIPPV. Data on demographics, APACHE III scores, degree of hypoxemia, ALI risk factors and NIPPV respiratory parameters were recorded. Univariate and multivariate regression analyses were performed to identify risk factors for NIPPV failure.

**Results:**

Of 79 consecutive patients who met the inclusion criteria, 23 were excluded because of a do not resuscitate order and two did not give research authorization. Of the remaining 54 patients, 38 (70.3%) failed NIPPV, among them all 19 patients with shock. In a stepwise logistic regression restricted to patients without shock, metabolic acidosis (odds ratio 1.27, 95% confidence interval (CI) 1.03 to 0.07 per unit of base deficit) and severe hypoxemia (odds ratio 1.03, 95%CI 1.01 to 1.05 per unit decrease in ratio of arterial partial pressure of O_2 _and inspired O_2 _concentration – PaO_2_/FiO_2_) predicted NIPPV failure. In patients who failed NIPPV, the observed mortality was higher than APACHE predicted mortality (68% versus 39%, *p *< 0.01).

**Conclusion:**

NIPPV should be tried very cautiously or not at all in patients with ALI who have shock, metabolic acidosis or profound hypoxemia.

## Introduction

Non-invasive positive pressure ventilation (NIPPV) is the accepted initial mode of treatment in subsets of patients with acute respiratory failure, the foremost exacerbation of chronic obstructive pulmonary disease with hypercarbia [1], and also in immunocompromised hosts [2,3], patients with cardiogenic pulmonary edema [4,5] and as a weaning aid in chronic obstructive pulmonary disease [6]. The efficacy of NIPPV in the initial management of other forms of hypoxemic respiratory failure, such as acute lung injury (ALI), pneumonia or postextubation respiratory failure, remains controversial [7-9]. Continuous positive airway pressure (CPAP) has been shown to be of no benefit in non-selected patients with acute hypoxemic respiratory failure and was associated with a higher number of adverse events [10]. While a prospective multicenter study identified ALI as an independent predictor of failure of NIPPV [11], specific underlying risk factors, such as presence of shock or metabolic acidosis, have not been evaluated in this group of patients. The uncertainty of the benefit of NIPPV in patients with ALI is reflected in a recent survey of NIPPV practice in which less than 40% of providers consider NIPPV to be beneficial in this group of patients [12]. The present study was undertaken to evaluate the outcome of patients with ALI treated with NIPPV as the initial mode of therapy and to identify factors predicting success/failure of NIPPV in this group of patients.

## Materials and methods

The present study was undertaken in two intensive care units (ICUs) of a tertiary care center. The institutional review board waved the informed consent requirement. Consecutive critically ill medical patients who met ALI criteria and who were treated with NIPPV as the initial mode of therapy between March and October of 2004 were included. The decision to intubate was left to the discretion of the treating intensivist. ALI was defined according to the American-European Consensus Conference definition [13]. Sepsis and shock were defined according to the American College of Chest Physicians/Society of Critical Care Medicine Consensus Conference definition [14].

**Figure 1:**
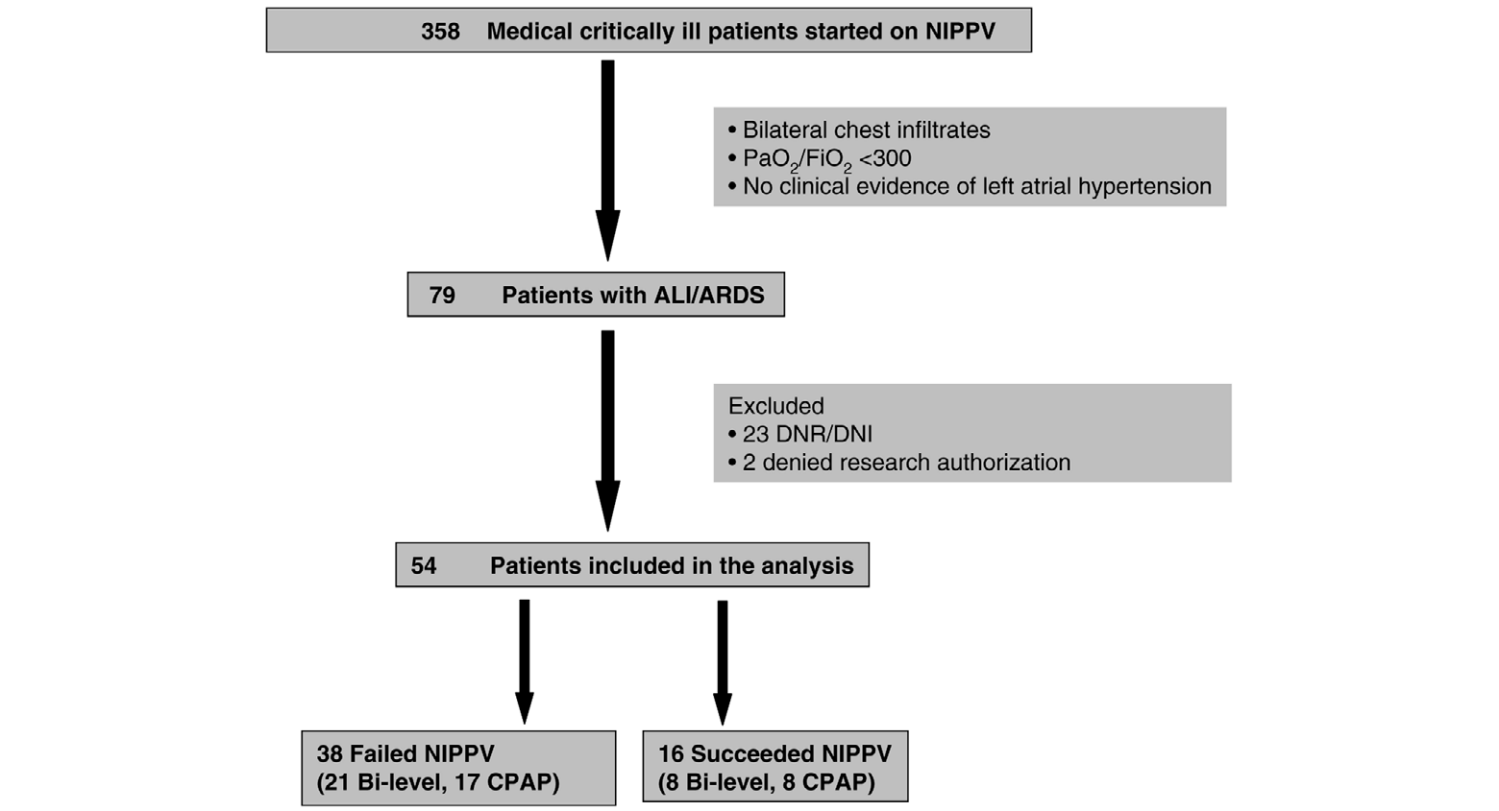
Outline of the study. CPAP, continuous positive airway pressure; DNI, do not intubate; DNR, do not resuscitate; NIPPV, non-invasive positive pressure ventilation. PaO_2_/FiO_2_, ratio of arterial partial pressure of O_2 _and inspired O_2 _concentration

Patients who had 'do not resuscitate/do not intubate' preferences (DNR/DNI) or refused research authorization were excluded (Figure 1). The main outcome measure was failure of NIPPV defined as subsequent intubation and invasive mechanical ventilation. Secondary outcomes were hospital mortality and ICU length of stay.

Data on demographics, DNR status, diagnoses, acute physiology parameters (vital signs, arterial blood gases, blood urea nitrogen, creatinine, bilirubin and hematocrit), severity of illness scores, mortality and length of ICU stay were prospectively collected by the bedside nurse into the Acute Physiology and Chronic Health Assessment (APACHE) III database. The characteristics of the ICU and the APACHE database have been previously described [15].

NIPPV was delivered through a full face mask in all patients. Patients who received bi-level pressure ventilation were ventilated with the 'Vision' NIPPV ventilator (Respironics Inc., Carlsbad, CA, USA). Data on inspiratory and expiratory pressure and estimated tidal volume were prospectively collected four times a day and documented in the respiratory therapy electronic medical record. The respiratory therapist confirmed the absence of air leak prior to recording the tidal volume value. The minority of patients in whom the initial mode of ventilation was CPAP, positive pressure was delivered by either 'Vision' NIPPV ventilator or a custom CPAP delivery system (Down's Flow Generator, Vital Signs Inc., Totowa, NJ, USA). In this group of patients the respiratory rate and airway pressure but not tidal volume were recorded.

Categorical variables were compared using standard Chi square and Fisher's exact tests as appropriate. Wilcoxon rank sum test was used to compare continuous variables. To evaluate the risk factors for NIPPV failure, a multivariate logistic regression model was created. Variables that were associated with NIPPV failure in univariate analysis (*p *< 0.1) were entered and a forward selection process identified the final model containing no more than three predictor variables. JMP statistical software (JMP, Version 5, SAS Institute Inc., Cary, NC, USA) was used for all data analyses.

## Results

A total of 358 patients received NIPPV over the six month study period. Of these, 79 patients initially started on NIPPV fulfilled criteria for ALI (Figure 1). After excluding 23 patients with DNR/DNI orders and two patients who refused research authorization, 54 patients were included in the analysis. The principal diagnosis was pneumonia in 34 (63%) patients, followed by vasculitis in 8 (15%) patients, nonpulmonary sepsis in 6 (11%) patients and interstitial lung disease exacerbation in 6 (11%) patients; 38 (70.3%) patients failed NIPPV. Table 1 describes clinical characteristics of the ALI patients sorted by outcome of NIPPV. Patients who failed NIPPV were more likely to have metabolic acidosis, higher severity of illness scores and a greater degree of hypoxemia (Table 1). None of the 19 patients with shock succeeded on NIPPV therapy. Being started on CPAP as opposed to bi-level ventilatory support as the form of NIPPV did not predict failure of therapy. The patients who failed NIPPV had higher minute ventilation and tended to have higher estimated tidal volumes (Table 2).

In a multivariate logistic regression analysis restricted to patients without shock, forward selection identified three variables (ratio of arterial partial pressure of O_2 _and inspired O_2 _concentration – PaO_2_/FiO_2_, base excess and APACHE III scores) in the final model (r^2 ^= 0.37). Metabolic acidosis (odds ratio 1.27, 95% confidence interval (CI) 1.03 to 0.07 per unit of base deficit) and severe hypoxemia (odds ratio 1.03, 95%CI 1.01 to 1.05 per unit decrease in PaO_2_/FiO_2_), but not APACHE III scores (odds ratio 1.46 95%CI 0.96 to 2.47), remained significant predictors of NIPPV failure. The median ICU length of stay in patients who failed NIPPV was significantly longer than in those who succeeded (median 8.9, 95% CI 4 to 13.2 days versus median 3, 95% CI 1.8 to 4.4 days, *p *< 0.01). In patients who failed NIPPV, the observed mortality was significantly higher than the APACHE III predicted mortality (68% versus 39%, p < 0.01). On the contrary, no deaths were observed in patients who succeeded NIPPV even though their predicted mortality approached 21%.

## Discussion

In our cohort of medical ICU patients with ALI who received NIPPV as the initial mode of therapy, two-thirds failed the NIPPV attempt. Our study confirmed expert recommendations that NIPPV should not be used in patients with hemodynamic instability [16]. In addition, presence of severe hypoxemia and metabolic acidosis were associated with NIPPV failure. While none of the patients who succeeded NIPPV treatment died in the hospital, the hospital mortality of patients who failed NIPPV was twice as high as predicted by the APACHE III score.

Hemodynamic instability is commonly cited as a contraindication to NIPPV by expert panels [16]; however, we did not find a specific clinical study on which this recommendation is based. Indeed, in a randomized study by Ferrer and colleagues [17], 12% of the enrolled patients were in shock when they were started on NIPPV. In our study, 35% of the patients had shock at the initiation of NIPPV, all of whom failed, that is, progressed to invasive mechanical ventilation. Moreover, the presence of metabolic acidosis was also predictive of NIPPV failure even when shock was not present at the time NIPPV was initiated. Although patients who failed NIPPV had a trend to have a higher level of serum lactate, this did not reach a statistically significant value, suggesting that not only tissue hypoperfusion, but metabolic acidosis *per se*, may be associated with poor outcome of NIPPV.

The risk of iatrogenic complications associated with NIPPV is thought to be lower than that associated with invasive mechanical ventilation. Interestingly, patients who failed NIPPV had larger tidal volumes and respiratory rates than those who succeeded (Table 1), in spite of somewhat lower inspiratory pressures than described in previous studies. Of note, large spontaneous tidal volumes and high respiratory rates may contribute to the development of permeability pulmonary edema as elegantly shown by Mascheroni and colleagues [18] in a sheep model. It is unclear, however, if breathing with large tidal volumes, which is a major risk factor for ventilator induced lung injury in intubated patients, confers the same risk in patients receiving NIPPV. Alternatively, the patients with metabolic acidosis or those with more severe lung injury and larger dead space required higher minute ventilation. Festic and colleagues [19] recently reported that delaying intubation in non-invasively ventilated patients with Pneumocystis pneumonia is associated with increased mortality. Among patients who failed NIPPV in the present study, non-survivors tended to have a longer delay in the intubation (14.5 hours versus 10.7 hours; *p *= not significant). However, the hypothesis that these patients would have benefited from earlier intubation and lung protective mechanical ventilation requires proof in a randomized controlled trial. On the other hand, it is important to emphasize that patients who succeeded NIPPV experienced better than predicted outcomes. The benefit of NIPPV seems to stem from avoiding the complications of intubation, including the increased need for sedation and the risk of ventilator associated pneumonia [3,20].

Our study confirmed the importance of severe hypoxemia identified in the previous multicenter study by Antonelli and colleagues [11]. Patients in our study who demonstrated improvement in PaO_2_/FiO_2 _after the NIPPV tended to have better outcome; however, this did not reach statistical significance (Table 2).

This study was performed at a single tertiary care center and the findings may not be generalizable. The observational nature of the study does not allow estimation of the cause and effect relationship between the predictors and outcome as unmeasured confounding factors may not have been accounted for. The decision to intubate was left to treating physicians and was not uniform or prospectively defined. Therefore, the threshold for intubation was probably lower in hypotensive hypoxic patients. The small sample size precluded adjusted analysis of NIPPV parameters. Although no patient was intubated because of intolerance to NIPPV interface *per se*, it is possible that suboptimal interface contributed to a failure in some patients. Furthermore, the accuracy of tidal volume estimates displayed on non-invasive mechanical ventilators has not been independently verified.

## Conclusion

We have observed a high failure rate of the initial NIPPV therapy in medical critically ill patients with ALI. Unless the underlying shock, metabolic acidosis and severe hypoxemia are rapidly resolved, a trial of NIPPV is unlikely to be successful. Given the higher than expected mortality in patients who failed a trial of NIPPV, it should be instituted with extreme caution in ALI patients who have shock, metabolic acidosis or severe hypoxemia.

## Key messages

• 	Hemodynamic instability and shock are major contraindications to non-invasive ventilation in patients with ALI.

• 	Metabolic acidosis and severe hypoxemia are associated with failure of non-invasive ventilation in patients with ALI.

• 	The hypothesis that high spontaneous tidal volumes may contribute to poor outcome of patients with ALI who are initially treated with non-invasive ventilation needs to be tested in prospective clinical trials.

• 	Carefully selected patients with ALI are successfully treated with non-invasive ventilation andtheir outcome is better than predicted by initial severity of illness.

## Abbreviations

ALI = acute lung injury; APACHE = Acute Physiology and Chronic Health Assessment; CI = confidence interval; CPAP = continuous positive airway pressure; DNR/DNI = do not resuscitate/do not intubate; ICU = intensive care unit; NIPPV = non-invasive positive pressure ventilation.

## Competing interests

The authors declare that they have no competing interests.

## Authors' contributions

SR, data collection, design, presentation. HJ, data collection, presentation. PCG, data analysis, presentation, discussion. CFB, data collection, design. RDH, data analysis, presentation, discussion. OG, design, analysis, presentation.

## Figures and Tables

**Table 1:** Clinical characteristics of medical ICU patients with ALI receiving NIPPV.

	NIPPV failure (*n *= 38)	NIPPV success (*n *= 16)	*p *value
Age	64 (54.7–73.2)	60 (46.2–83.7)	0.86
Female gender	13 (34%)	8 (50%)	0.27
APACHE III score	81.5 (64–103.5)	55.5 (43–73.75)	<0.01
SOFA score	8.5 (6–11)	5.5 (3.25–7.75)	<0.01
Sepsis	33 (87%)	14 (88%)	0.90
Shock	19 (100%)	0 (0%)	-
Transfusion	11 (29%)	5 (31%)	0.80
Aspiration	14 (37%)	6 (38%)	0.90
Immunosuppression	18 (47%)	5 (31%)	0.37
PaO_2_/FiO_2_	112 (70–157)	147 (118–209)	0.02
PaCO_2_	36 (32–44)	42 (36–48)	0.10
pH	7.37 (7.26–7.43)	7.39 (7.32–7.45)	0.40
Base excess	-4.0 (-7–0.02)	0.5 (-3–1.3)	0.01
Lactate (*n *= 37)	2.1 (1.2–4.0)	1.5 (1.2–2.1)	0.26

ALI, acute lung injury; APACHE, Acute Physiology and Chronic Health Assessment; ICU, intensive care unit; NIPPV, non-invasive positive pressure ventilation. PaO_2_/FiO_2_, ratio of arterial partial pressure of O_2 _and inspired O_2 _concentration; SOFA, Sequential Organ Failure Assessment, PaCO_2_, arterial partial pressure of CO_2_

**Table 2:** Non-invasive positive pressure ventilation parameters

	NIPPV failure (*n *= 38)	NIPPV success (*n *= 16)	*p *value
Initial mode of ventilation			
Bi-level NIPPV	21	8	0.72
CPAP	172	84	
Respiratory rate (initial; breaths/minute)	31 (24–39)	23 (21–34)	0.13
Tidal volume (ml; *n *= 27)	640 (493–700)	435 (384–558)	0.03
Minute ventilation (l/minute; *n *= 27)	17.2 (12–27)	12.5 (8–14)	0.02
Tidal volume (ml/kg predicted body weight; *n *= 27)	9 (7.5–11)	8.4 (6.4–10.4)	0.34
IPAP (cmH_2_O; *n *= 29)	12 (10–16)	13 (12–15)	0.53
EPAP or CPAP (cmH_2_O; *n *= 43)	5 (5–7.5)	5.5 (5–8)	0.92
Decrease in respiratory rate after NIPPV^a^	13 (34%)	9 (56%)	0.24
Improvement in arterial blood gas PaO_2_/FiO_2 _after the NIPPV^b^	7 (18%)	6 (38%)	0.17

^a^Respiratory rate <30 and a 10% decrease from before the non-invasive positive pressure ventilation (NIPPV) institution. ^b^Improvement in PaO_2_/FiO_2 _>10 units after the start of NIPPV (within one hour). Patients who were intubated before the follow up blood gas measurement were considered not to have improvement. APACHE, Acute physiology and chronic health evaluation; CPAP, continuous positive airway pressure; EPAP, expiratory positive airway pressure; IPAP, inspiratory positive airway pressure. PaO_2_/FiO_2_, ratio of arterial partial pressure of O_2 _and inspired O_2 _concentration;
